# Polarity-considered EEG microstates improve classification accuracy of oddball stimulus

**DOI:** 10.3389/fnhum.2026.1712380

**Published:** 2026-03-18

**Authors:** Tatsumi Tsubaki, Shiho Kashihara, Tomohisa Asai, Hiroshi Imamizu, Isao Nambu

**Affiliations:** 1 Graduate School of Engineering, Nagaoka University of Technology, Niigata, Japan; 2 Department of Cognitive Neuroscience, Cognitive Mechanisms Laboratories, Advanced Telecommunications Research Institute International (ATR), Kyoto, Japan; 3 Department of Psychology, Graduate School of Humanities and Sociology, The University of Tokyo, Tokyo, Japan

**Keywords:** BCI, classification, EEG microstates, labeling, oddball task

## Abstract

Brain–computer interfaces (BCIs) require efficient feature extraction and dimensionality reduction from high-dimensional neural signals. Electroencephalogram (EEG) microstate analysis is a rapid and noise-resistant approach that classifies instantaneous EEG states into several spatial distribution patterns (templates). Previous BCI studies using the EEG microstate approach have typically used aggregated metrics, such as duration, frequency of occurrence, or time coverage, and have rarely applied pointwise microstate labeling as temporally ordered, one-dimensional sequences for robust classification. Moreover, the physiological relevance of EEG topographic polarity has often been overlooked, despite its potential to reveal smoother state transitions and align with event-related potential components. In this study, we applied polarity-considered microstate labeling to stimulus-driven classification in an oddball paradigm. EEG data from 40 healthy participants (20 per response type) were analyzed across three factors: stimulus modality (auditory or visual), modality condition (unimodal or cross-modal), and response type (key-response task or mental counting task). Preprocessed 32-channel EEG data were labeled with microstate templates (A–E ± topographical polarity) using a winner-take-all approach, and the resulting sequences were classified using multiple machine-learning models. The results showed that tree-based ensemble models (Random Forest, XGBoost, and CatBoost) achieved the most stable and accurate performance in the key-response task with cross-modal visual targets. These models reached an area under the receiver operating characteristic curve above 0.8 and a mean F1 score of 0.83. Preserving polarity improved classification by approximately 20% across tasks, doubling the label-space granularity and revealing temporal patterns aligned with the N200 and P300 components. Visual stimuli generally outperformed auditory stimuli, and cross-modal benefits emerged primarily in key-response tasks. These findings demonstrate that polarity-considered microstate labeling enhances classification accuracy and interpretability in BCIs. This method highlights the potential for real-time applications, such as P300 spellers and multimodal attention monitoring.

## 1 Introduction

Brain–computer interface (BCI) systems analyze brain signals to control external devices and are promising for a wide range of applications, including medical care and daily life support. A key requirement for BCI implementation is the extraction and dimensionality reduction of the features from high-dimensional brain signals. The P300 speller is a character input system that utilizes event-related potentials (ERPs) elicited by focusing attention on flashing characters, as first proposed by Farwell and Donchin (1988). Although BCI systems are considered practical for individuals with severe motor impairments such as those with amyotrophic lateral sclerosis, they have not yet achieved widespread usability. A major barrier is the limited classification performance and detection accuracy of the P300 component in single-trial analyses. Farwell and Donchin (1988) identified the Pz electrode (located over the parietal region) as the primary site for observing the P300 component, and Rezeika et al. (2018) emphasized its parietal predominance. However, Krusienski et al. (2008) demonstrated that using several electrodes improved classification accuracy. These findings suggest that expanding spatial coverage can enhance P300 detection performance.

Electroencephalogram (EEG) microstate analysis (Lehmann et al., 1987; Michel and Koenig, 2018) classifies multi-channel EEG signals into spatial patterns (templates) and extracts the characteristics of each template as features. This approach facilitates the interpretation of brain activity during task performance. Moreover, employing microstate analysis with many electrodes enhances the interpretability of brain states involved in EEG-based classification. This study applied EEG microstate analysis in a BCI system to detect P300 components and extract features reflecting more comprehensive brain dynamics.

Existing BCI studies utilizing EEG microstate analysis (Cui et al., 2023; Xiong et al., 2024; Wang et al., 2023; Kim and Kim, 2020; Zhao et al., 2025) have typically generated four to five microstate templates and extracted low-dimensional features such as mean duration, frequency of occurrence, time coverage, and transition probability. Cui et al. (2023) examined inter-subject variability in motor imagery BCI performance by analyzing four types of EEG microstates using the above metrics. However, to the best of our knowledge, EEG microstate labeling has not been previously applied to BCI systems, in which each EEG time point is assigned to the template with the highest spatial correlation. Although Zhao et al. (2025) employed sequence-to-sequence deep learning to predict microstate transitions for online applications, their approach targeted temporal forecasting rather than stimulus classification. The EEG microstate labeling approach enables the extraction of one-dimensional features that are computationally efficient and robust to noise, facilitating the practical deployment of future BCI systems.

Previous studies (Lehmann et al., 1987; Michel and Koenig, 2018; Cui et al., 2023; Xiong et al., 2024; Wang et al., 2023; Kim and Kim, 2020; Zhao et al., 2025) have typically ignored the polarity (positive/negative) of EEG signals in microstate analyses. However, Kashihara et al. (2025a) proposed that incorporating polarity into microstate labeling would enable smoother transitions and improve the detection of age-related changes in brain dynamics. Based on this insight, we hypothesized that incorporating polarity into microstate templates by adding polarity-inverted versions of the existing templates would allow the extraction of more informative features. Mahini et al. (2024) suggested that the topographical changes observed in the oddball response, particularly components such as N200 and P300, may reflect polarity-inverted isomorphic states. Therefore, this study introduces polarity-considered microstate labeling to achieve more effective feature extraction. Kashihara et al. (2025a) suggested that polarity reflects neural sources and structural constraints. Hence, the inclusion of polarity may allow microstates to capture neural activity with greater physiological significance. In addition, it is important to examine differences between conventional ERP measures based on raw EEG signals and ERP-like representations derived from microstate labeling. To this end, this study compared microstate sequences across stimulus conditions and analyzed time-resolved microstate label distributions. These analyses aimed to visualize structural differences in stimulus response patterns and identify brain states associated with successful recognition.

The oddball paradigm (Squires et al., 1975) is a widely used cognitive task for studying P300 responses in BCI research. This paradigm randomly presents infrequent (“target”) stimuli among frequent (“standard”) stimuli. We explored stimulus classification within the oddball paradigm to develop a BCI system incorporating polarity-considered microstate labeling for improved classification accuracy. We hypothesized that refining the task environment would lead to further improvements in performance. Kashihara et al. (2025b), used three experimental factors in the oddball paradigm: (i) the modality of infrequent stimuli (auditory or visual), (ii) the combinations of frequent and infrequent stimuli (unimodal or cross-modal), and (iii) the type of response to infrequent stimuli (key-response task or count task). Existing studies are inconsistent regarding whether visual (Katayama and Polich, 1999; Polich and Heine, 1996) or auditory stimuli (Bennington and Polich, 1999) elicit stronger responses. Research on stimulus combinations (Brown et al., 2006; Brown et al., 2007) suggests that late components, including the P300, tend to be enhanced under cross-modal conditions, whereas early components remain unaffected. Regarding the response type, some studies have reported enhancement of the P300 with key-response tasks (Brázdil et al., 2003; Kotchoubey, 2014), others with count tasks (Barrett et al., 1987; Salisbury et al., 2001), and others have reported no significant difference (Starr et al., 1995). Nevertheless, from the perspective of classification performance in oddball-based BCIs, cross-modal combinations of frequent and infrequent stimuli offer potential advantages. These experimental factors are known to influence ERP components, and evidence suggests that polarity-considered microstate labeling can capture similar brain responses (Kashihara et al., 2025b). Accordingly, applying polarity-considered microstate labeling to classification tasks under varied experimental conditions may help identify optimal task settings for this method.

Therefore, the present study focused on the following three objectives: (I) to evaluate the classification performance of P300 responses using microstate labeling, which differs from conventional approaches; (II) to investigate the potential improvement in performance achieved by utilizing polarity-considered microstate labeling, a recent advancement in the field; (III) to examine the correspondence between classification performance under different experimental conditions in the oddball paradigm and the application of these labeling methods. For objective (I), we evaluated whether the classification performance using one-dimensional microstate sequences exceeded the chance levels. Although linear classifiers, such as linear discriminant analysis and step-wise linear discriminant analysis (SWLDA), have been widely adopted for P300 classification, with SWLDA achieving over 90% accuracy in previous studies (Krusienski et al., 2008), this study compares a broader set of models (Support Vector Machine: SVM, Random Forest, Logistic Regression, XGBoost, CatBoost, and K-means). To achieve objective (II), we evaluated whether polarity-considered labeling performed better than conventional polarity-ignored labeling. Additionally, we explored the neuroscientific underpinnings of this approach from the perspective of topographical dynamics of brain activity. For objective (III), we investigated whether known differences in ERP responses under various oddball conditions, such as stimulus modality, frequency ratio, and response type, were reflected in the classification performance using polarity-considered microstate labeling. Based on these objectives, we discuss the implications of our results for future applications in BCI system development.

## 2 Materials and methods

### 2.1 Experiment data

For classification, we used data from the oddball task conducted by Kashihara et al. (2025b). Two datasets were collected from 20 healthy participants in their 20s and 30s. These datasets were approved by the Ethics Committee of ATR (approval numbers: 21–144 and 21–143). The experiment was designed to examine the three factors described in Section 1. Four stimulus conditions were used: auditory only (unimodal auditory: uniA), visual only (unimodal visual: uniV), high-frequency visual with low-frequency auditory (cross-modal auditory target: croA), and high-frequency auditory with low-frequency visual (cross-modal visual target: croV). The modality conditions were counterbalanced across participants. Auditory stimuli consisted of pure tones at 1,000 Hz for standard stimuli and 2,000 Hz for target stimuli. The visual stimuli consisted of circles as the standard stimuli and stars as the target stimuli. Two task groups were defined for responses to target stimuli: the key-response task (keyresTask), in which participants (all right-handed) were instructed to press the spacebar with their right index or middle finger as quickly and accurately as possible, and the counting task (countTask), in which participants silently counted the number of target stimuli. In the countTask, stimulus frequency assignments were counterbalanced across participants. The keyresTask group included 12 females and eight males (mean age = 30.7 years, SD = 6.9), and the countTask group included 10 females and 10 males (mean age = 25.0 years, SD = 5.3). EEG recordings were performed using an R-Net 32-channel system (Brain Products GmbH, Gilching, Germany) and BrainAmp MR plus amplifier (Brain Products GmbH). The 32 electrode positions followed the international 10–10 system (Fp1, Fp2, Fz, F3, F4, F7, F8, F9, F10, FC1, FC2, FC5, FC6, Cz, C3, C4, T7, T8, CP1, CP2, CP5, CP6, Pz, P3, P4, P7, P8, P9, P10, Oz, O1, and O2). The EEG sampling rate was set to 500 Hz for the keyresTask and 5,000 Hz for the countTask. Each trial consisted of a 200 ms stimulus presentation and a 1,000 ms interstimulus interval. A total of 320 standard and 80 target stimuli were presented, with target stimuli pseudo-randomized to avoid consecutive occurrences. In keyresTask, trials containing errors, either false alarms (incorrect key press) or misses (no response), were excluded from the analysis. After artifact rejection, the minimum numbers of valid standard and target trials across participants were 312 and 68, respectively. Further details can be found in a study by Kashihara et al. (2025b).

### 2.2 Preprocessing

The EEG signals acquired in the experiment were resampled at 1000 Hz. A bandpass filter from 1 to 45 Hz was then applied using a finite impulse response filter. Subsequently, bad-channel detection and rejection as well as Artifact Subspace Reconstruction (ASR) (Mullen et al., 2015) were performed using the EEGLAB clean_rawdata plugin. EEG channels were identified as bad and removed when any of the following criteria were satisfied (keyresTask: mean number of removed channels = 1.50, SD = 1.54; countTask: mean number of removed channels = 1.83, SD = 1.63): a flat signal persisting for more than 5 s, line noise exceeding 4 standard deviations relative to the total channel signal, or a correlation coefficient with neighboring channels of less than 0.85. Subsequently, ASR was applied to reconstruct nonstationary noise. Sliding windows with RMS values exceeding 10 standard deviations relative to an automatically identified clean calibration dataset were reconstructed based on the remaining components. Next, the data were re-referenced to the average reference, after which Independent Component Analysis (ICA) was performed using Adaptive Mixture ICA (AMICA) (Palmer et al., 2008). Equivalent current dipoles were then estimated for each independent component (IC) using the DIPFIT plug-in, followed by bilateral dipole fitting using the fitTwoDipoles plug-in (Piazza et al., 2016). ICs were further classified using ICLabel (Pion-Tonachini et al., 2019). ICs were retained only when labeled as “brain” by ICLabel, exhibiting a dipole residual variance of less than 15% (Artoni et al., 2014), and localized within brain regions; all other ICs were removed. In addition, ICs with amplitudes exceeding 150 μV following ICA decomposition were rejected as artifactual components. This procedure resulted in an average of 12.63 retained ICs (SD = 3.37) in keyresTask and 13.60 ICs (SD = 3.26) in countTask. The EEG data were then segmented into epochs ranging from 200 ms before to 1,000 ms after the stimulus onset. All preprocessing steps prior to epoching were performed in MATLAB (R2019a, MathWorks, Natick, MA, United States) using EEGLAB (v2019.1) (Delorme and Makeig, 2004) and its associated plugins.

**Figure 1:**
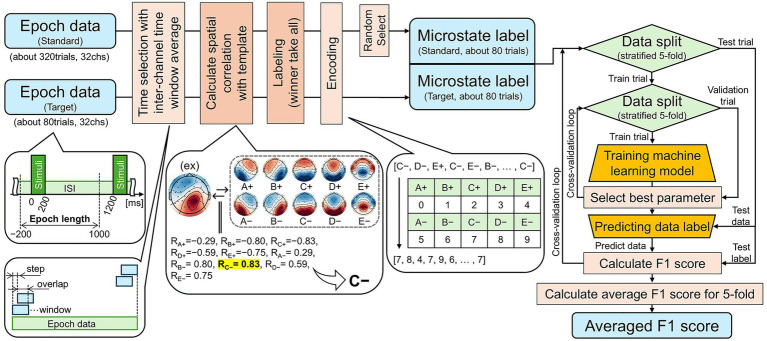
Overview of the encoding and classification processes, where encoding denotes the transformation of EEG epochs (−200 to 1,000 ms relative to stimulus onset) into one-dimensional microstate label sequences, and classification denotes the binary discrimination of standard versus target stimuli in the oddball paradigm using these labeled sequences. The EEG topographies shown in the center represent a set of 10 microstate templates derived from the LEMON dataset (Babayan et al., 2019), obtained using modified K-means clustering with five clusters and their corresponding polarity-inverted maps. The resulting one-dimensional microstate label sequences (right) were used as inputs to machine learning models for stimulus classification. Model performance was evaluated using five-fold cross-validation, and F1 scores were computed from the predicted and true labels, then averaged across folds.

An overview of the post-epoch data processing pipeline is shown in Figure 1. The channel-wise mean was computed within the specified time windows. The resulting spatial distribution at each time point was correlated with the templates provided by Kashihara et al. (2025a), and the template with the highest correlation was assigned using a winner-take-all approach. The templates created by Kashihara et al. (2025a) were derived from the LEMON dataset (Babayan et al. 2019) using modified K-means clustering, resulting in five spatial maps. By including polarity-inverted versions of each, a total of 10 microstates (A± to E±) were obtained (Figure 1, center left). The template-matching results were then encoded as integers from zero to nine. Finally, a random subset of standard trials was selected to balance the dataset and equalize the number of trials between the standard and target stimuli.

### 2.3 Label occurrence frequency

To investigate the temporal dynamics of the microstate labels obtained in Section 2.2, the occurrence frequency of each label was calculated within sliding time windows of 100 ms, advanced in 25 ms steps (i.e., 75% overlap). For each time window, the occurrence frequency was defined as the total count of each of the 10 template labels across all trials divided by the total number of trials.

### 2.4 Classification

#### 2.4.1 Temporal generalization matrix

To evaluate how temporal patterns generalize across trials, a temporal generalization matrix was constructed based on the processed data using a time window size of 100 ms, advanced in 25 ms steps (i.e., 75% overlap), following the approach of Veillette et al. (2023). To classify standard versus target stimuli in this matrix, a model was trained using a sliding window of three consecutive time points (i.e., a target time point and its immediate neighbors), and predictions were generated by shifting the test time window across time points. Classification performance was assessed using the F1 score (Equation 1).


$$F1\;\text{score}=\frac{2\cdot \text{Precision}\cdot \text{Recall}}{\text{Precision}+\text{Recall}}=\frac{2\mathrm{TP}}{2\mathrm{TP}+\mathrm{FP}+\mathrm{FN}}$$ (1)

$$\left(\text{Precision}=\frac{\mathrm{TP}}{\mathrm{TP}+\mathrm{FP}},\text{Recall}=\frac{\mathrm{TP}}{\mathrm{TP}+\mathrm{FN}}\right)$$

The F1 score represents the balance between precision and recall, which increases with a higher number of true positives (TP) and decreases when false positives (FP) or false negatives (FN) are increased. TP refers to correctly classified target stimuli; FP, standard stimuli misclassified as target; TN, correctly classified standard stimuli; FN, target misclassified stimuli. This matrix aims to identify the most suitable time periods for classification. While Veillette et al. (2023) used logistic regression to classify EEG signals related to the sense of agency during self-executed and unexecuted motor tasks based on electromyogram information, this study employed a SVM, which also allows for nonlinear classification, to evaluate performance. The SVM was implemented using the Python-based scikit-learn package (version 1.5.2). Its hyperparameters were optimized through a grid search on the validation dataset, testing C values of 0.01, 0.1, 1, 10, and 100; kernel types of linear, rbf, and sigmoid; and gamma options of scale, auto, and 1.

#### 2.4.2 Classification using six models

Based on the results in Section 2.4.1, the analysis was restricted to periods suitable for classification, and six different models were trained to predict whether each trial corresponded to a standard or target stimulus. The F1 score was used as an evaluation metric. For this analysis, the time window size was set to 10 ms, advanced in 1 ms steps (i.e., 90% overlap). Although this differs from the previous window size, a smaller window is expected to yield a higher temporal precision. The six classification models used were SVM, Random Forest, Logistic Regression, XGBoost, CatBoost, and K-means. These models were selected based on previous studies (Barrett et al., 1987; Salisbury et al., 2001; Starr et al., 1995), emphasizing those known for their high classification performance and computational efficiency. Model performance was evaluated using five-fold cross-validation at the trial level. For each fold, the training data were further split into training and validation sets using five-fold cross-validation to optimize the hyperparameters (Supplementary Table 1) using Python (version 3.12.7). The classifier was then retrained on the full training set using the best parameters, and predictions were made on the test set, which comprised 20% of the total data. The F1 scores of the five folds were averaged to obtain the final mean F1 score. The same analysis was performed for the polarity-ignored version.

#### 2.4.3 Evaluation

The performances of the classification models were evaluated using the area under the receiver operating characteristic curve (AUC). According to Hanley and McNeil (1982), AUC represents the probability that a randomly selected positive instance is ranked higher than a randomly selected negative instance, with 0.5 representing chance-level performance. To assess the effects of the three experimental factors described in Section 1, namely, the modality of target stimuli, the combination of standard and target stimuli, and the response type to target stimuli, a three-way mixed-design analysis of variance (ANOVA) was conducted. This design was appropriate because response type (keyresTask vs. countTask) constituted a between-subject factor, as separate participant groups performed each task, whereas the remaining factors were within-subject variables. This analysis used the average F1 score of the classification model that demonstrated the most stable and accurate AUC performance. To evaluate the influence of polarity on classification outcomes, we also calculated F1 scores using microstate templates that did not account for polarity. Furthermore, to examine whether the classification performance depended on the number of templates, we computed the F1 scores under three additional conditions: (1) using four templates, (2) using seven clusters derived in a data-driven manner (7_dat), and (3) using five clusters with two additional artificially created templates generated by averaging the centroids of microstates A and B and microstates D and E, respectively (7_art). The results were analyzed using ANOVA.

### 2.5 Time-resolved microstate label analysis

To examine temporal differences in microstate label distributions between standard and target stimuli, EEG data from −200 to 400 ms relative to stimulus onset were segmented using a 10 ms sliding window with 90% overlap, and microstate labeling was applied to each segment. The resulting labels were one-hot encoded and averaged across trials for each participant. Permutation-based t-tests with cluster correction were conducted at each time point across participants, with shuffling restricted to the temporal dimension. In addition, false discovery rate correction was applied to incorporate multiple comparisons across microstate labels. To assess whether such differences were linked to successful classification, the same procedure was repeated separately for correctly and incorrectly classified trials using a high-performing Random Forest model.

### 2.6 Topographic reconstruction via microstate weights

To evaluate potential information loss when reducing EEG signals to microstate label representations, we reconstructed the temporal evolution of EEG topographies based on the averaged microstate label distributions obtained in Section 2.5. At each time point, the one-hot-encoded microstate label averages were multiplied by the corresponding microstate template maps. The weighted linear sum across all templates was computed to reconstruct the EEG topography at each time point. To quantify the fidelity of this reconstruction, we calculated the Global Dissimilarity (DISS; Equation 2) between the reconstructed topographies and grand-averaged EEG signals (i.e., across all trials and participants), following the approach described by Murray et al. (2008).


$$\text{DISS}(u,v)={\left\|\frac{u}{{\left\|u\right\|}_{2}}-\frac{v}{{\left\|v\right\|}_{2}}\right\|}_{2}=\sqrt{\sum_{i=1}^{N}{\left(\frac{u_{i}}{{\left\|u\right\|}_{2}}-\frac{v_{i}}{{\left\|v\right\|}_{2}}\right)}^{2}}$$ (2)

In Equation 2, *u* represents the reconstructed EEG signals, *v* represents the grand-averaged EEG signals, *i* denotes the channel index, and *N* is the total number of channels. DISS is mathematically equivalent to the Global Map Dissimilarity (Lehmann and Skrandies, 1980), differing only by a scaling factor, and is defined as the Euclidean distance between normalized voltage maps. Importantly, this measure is equivalent to a rescaled spatial correlation between maps. Thus, DISS provides a scale-independent measure of topographic dissimilarity, ranging from zero (identical maps) to two (polarity-inverted maps), and reflects differences in the spatial configuration of scalp potentials rather than amplitude.

## 3 Results

### 3.1 Label occurrence frequency

**Figure 2:**
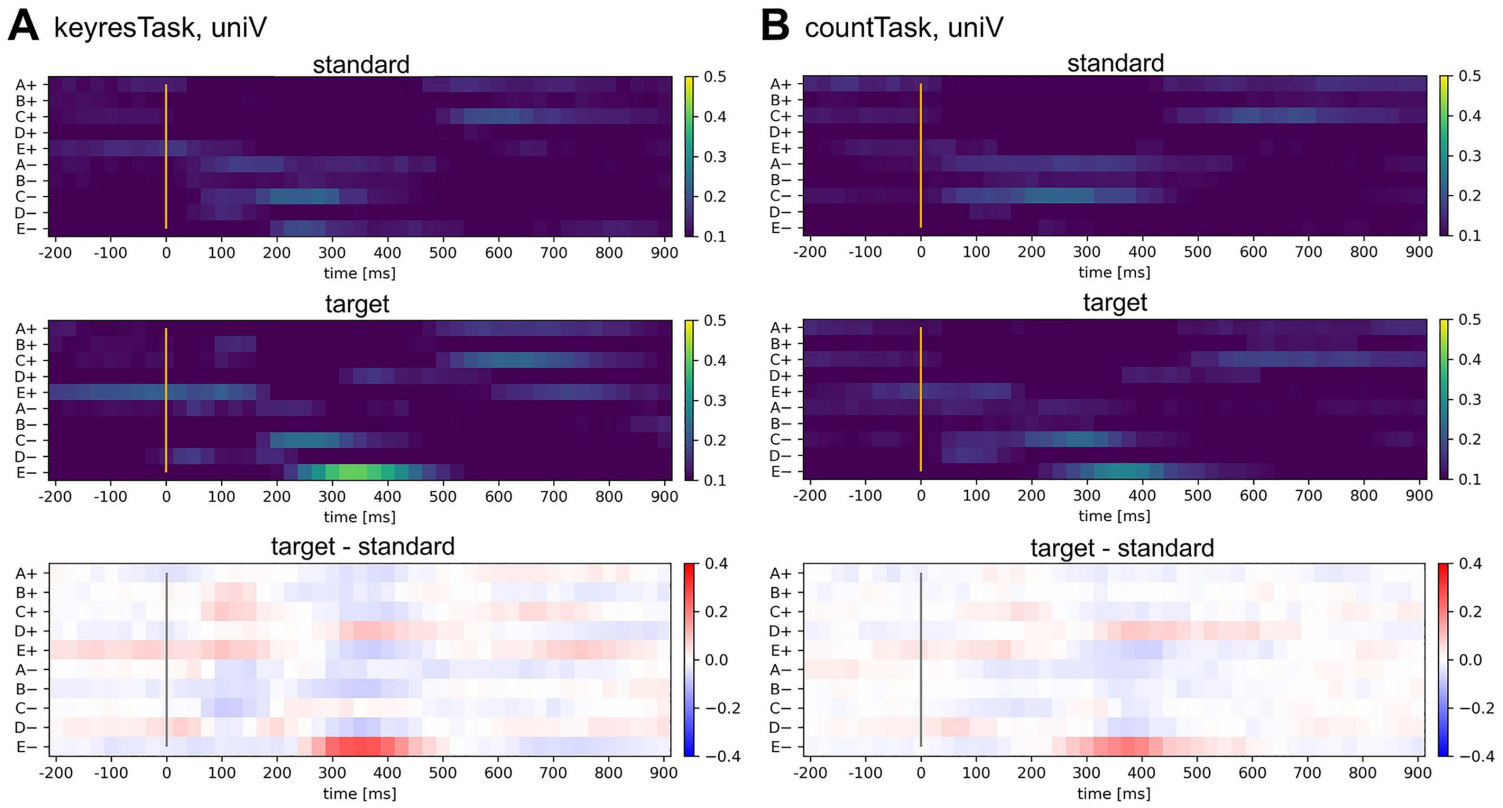
EEG microstate occurrence rates in the uniV condition for the keyresTask and countTask. **(A)** Results for the key-response task. From top to bottom: the occurrence rate during standard (frequent) stimuli, the occurrence rate during target (infrequent) stimuli, and the difference between target and standard stimuli. The horizontal axis represents time (ms), and the vertical axis indicates template labels. Color bars represent occurrence rates. For standard and target stimuli (top and middle panels), a sequential colormap was used, with higher values shown in warmer colors. For the difference map (bottom panel), a diverging colormap was applied, where red indicates higher occurrence during target stimuli relative to standard stimuli and blue indicates the opposite. **(B)** Results for the count task presented in the same format as in **(A)**.

Figure 2 shows the average label occurrence rates for EEG microstate labels across participants in the uniV-only condition. As shown in Figure 2A, during the keyresTask, target visual stimuli elicited higher occurrence rates of C+ and E+ than standard stimuli within the first 200 ms following stimulus onset. This was followed by a transition to D+, C−, and E− (300–400 ms) and a return to C+ and E+ after 500 ms. A similar pattern was observed for countTask (Figure 2B).

### 3.2 Result of temporal generalization matrix

**Figure 3:**
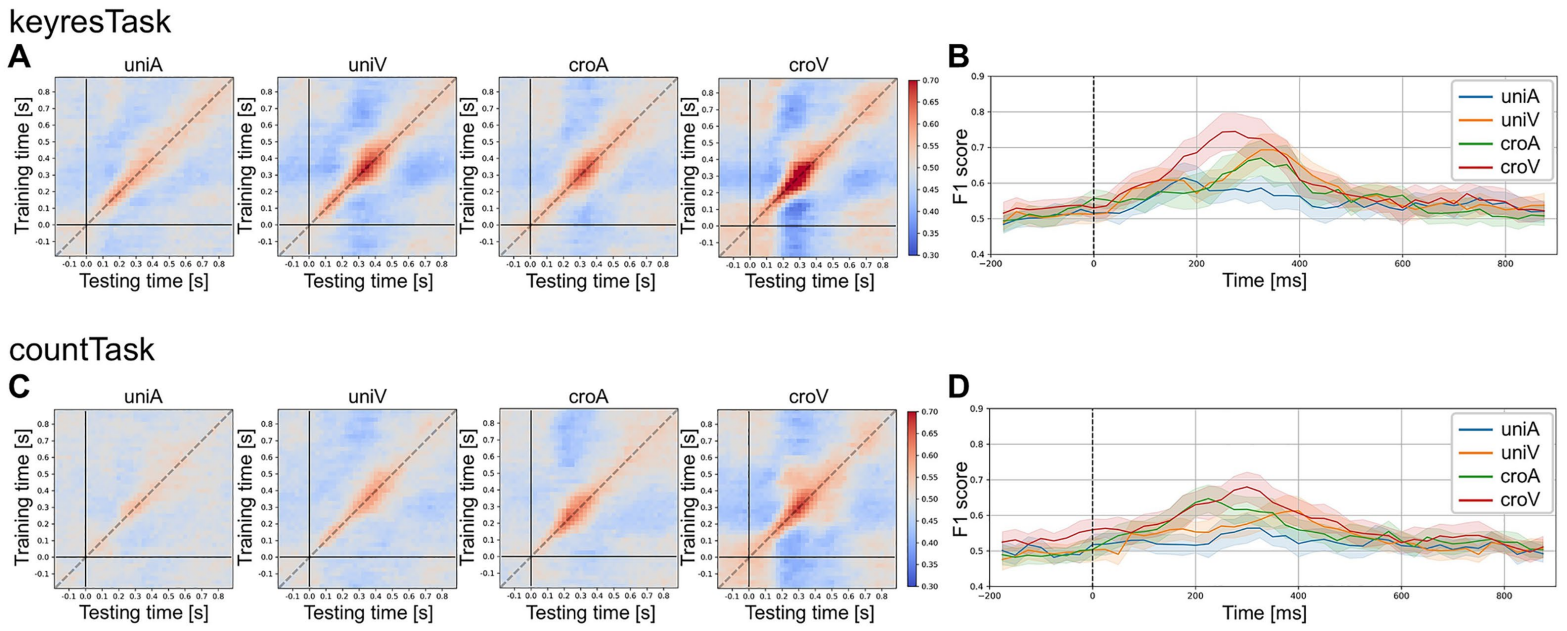
Temporal generalization matrices and time-resolved F1 scores. **(A,C)** Present the temporal generalization matrices averaged across all participants (*n* = 20) for the keyresTask and countTask, respectively (horizontal axis: testing time; vertical axis: training time). **(B,D)** Show the mean F1 scores and 95% confidence intervals across participants along the diagonal of the matrices shown in **(A,C)**, respectively.

The results of the temporal generalization matrix are shown in Figure 3. As shown in Figures 3A,C, F1 scores were low during the pre-stimulus period (−200 to 0 ms) and showed a clear diagonal pattern, with higher scores when the training and testing time points corresponded. Notably, training on data from the 0 to 250 ms range increased the F1 scores when testing on data after 500 ms. Conversely, training on the data after 500 ms improved classification for testing in the 0–250 ms range. Figures 3B,D show that regardless of response type, the croV condition yielded the highest F1 scores (keyresTask: F1 score at 275 ms = 0.745 ± 0.025; countTask: F1 score at 300 ms = 0.680 ± 0.020). By contrast, the uniA condition produced the lowest scores (keyresTask: F1 score at 175 ms = 0.615 ± 0.020; countTask: F1 score at 325 ms = 0.564 ± 0.013). Across all experiments, the average F1 scores across participants peaked in the 0–400 ms range. Therefore, the 0–400 ms time window was used in subsequent classification analyses.

### 3.3 Results of classification using six models

#### 3.3.1 Effects of stimulus modality, modality conditions, and response type

**Figure 4:**
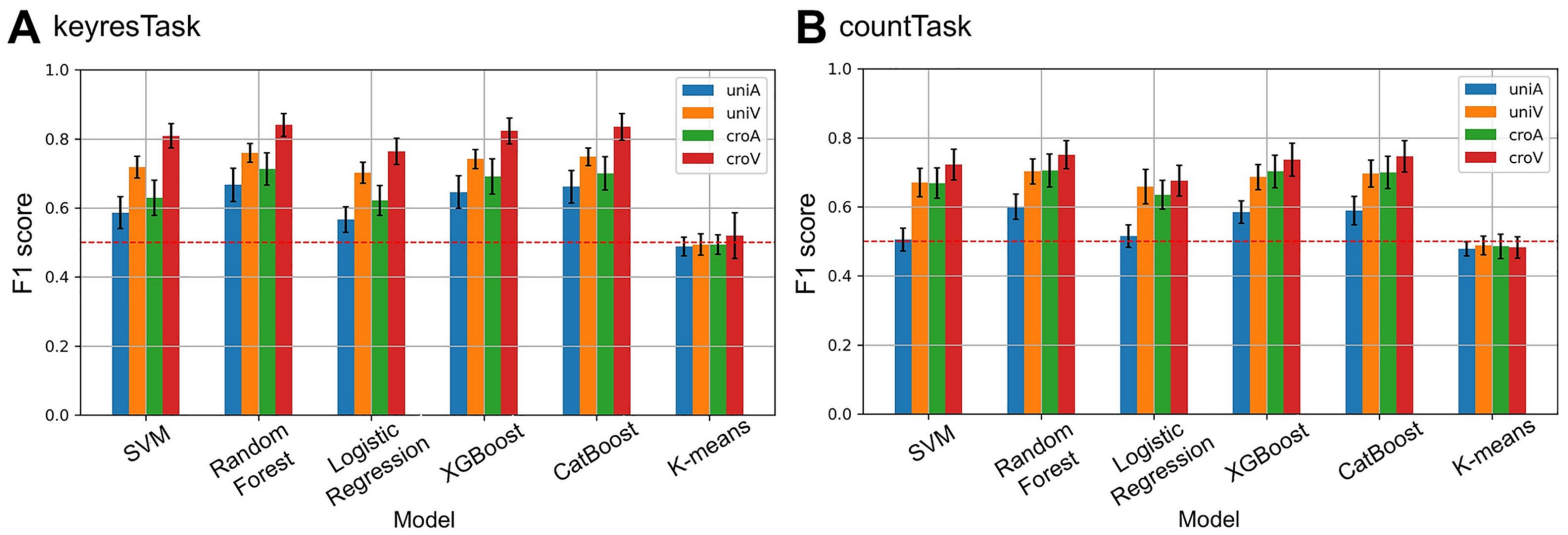
F1 scores for each machine learning model across task types and stimulus conditions. **(A)** Results from keyresTask. **(B)** Results from countTask. Color represents stimulus condition (blue: uniA, orange: uniV, green: croA, red: croV). Error bar indicates 95% confidence intervals across participants. The red dashed line represents the chance level (0.5). Across models, classification performance varied depending on task type and stimulus condition, with croV in keyresTask yielding the highest F1 scores. By contrast, K-means clustering remained approximately at the chance level across all conditions.

Based on the results in Section 2.4.2, six classification models were applied to the 0–400 ms time window, which showed higher F1 scores (Section 3.2). The results are shown in Figure 4. All models, except K-means, achieved classification performance above the chance level regardless of the response type, with the highest performance observed in the croV condition. However, the F1 scores for the SVM and Logistic Regression models were close to the chance level for the uniA condition in countTask.

**Figure 5:**
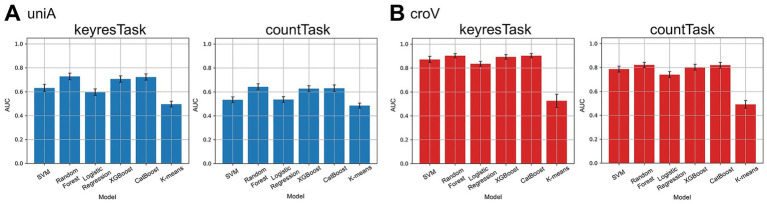
AUC bar plot for each machine learning model. **(A)** Results from the conditions with the lowest F1 scores (uniA), in both keyresTask and countTask. **(B)** Results from the conditions with the highest F1 scores (croV). The vertical axis represents the AUC value, providing an additional evaluation of classification performance across models. Among the classifiers, the tree-based models—Random Forest, XGBoost, and CatBoost—achieved the most stable and reliable performance.

Similar trends were observed across the five classification models, excluding K-means, for both the task types. A correspondence matrix was constructed to visualize these relationships (Supplementary Figure 1). Additionally, the AUC was calculated as a performance metric for each classification model. The AUC distributions for the highest-performing (croV) and lowest-performing (uniA) conditions are shown in Figure 5, where Random Forest, XGBoost, and CatBoost demonstrated the most stable and accurate classification performance. Notably, in keyresTask under the croV condition, the average AUC exceeded 0.8, even when SVM and Logistic Regression were included, indicating highly reliable classification results.

**Figure 6:**
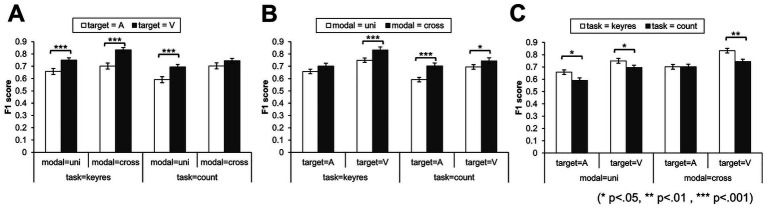
F1 scores for the three experimental factors in the oddball task. **(A)** Target stimulus modality (auditory vs. visual). **(B)** Modality combination (unimodal vs. cross-modal). **(C)** Response type (key-response vs. count). Error bar represents 95% confidence intervals across participants. Significant effects were primarily observed for visual stimuli in target modality factor, for cross-modal compared with unimodal conditions in the keyresTask with visual targets, and for the keyresTask compared with the countTask in conditions with auditory infrequent stimuli.

Based on the AUC values, a three-way mixed-design ANOVA was conducted using the average scores of the three decision-tree-based models (Random Forest, XGBoost, and CatBoost), which showed the most stable and accurate performance. The analysis revealed a significant second-order interaction effect [*F*(1, 38) = 4.88, *p* < 0.05, $\eta_{p}^{2}\;$ = 0.11]. With respect to the modality of target stimuli, visual stimuli yielded significantly higher f1 scores than auditory stimuli across all conditions except for the cross-modal condition in countTask (Figure 6A). For stimulus modality combinations, cross-modal conditions resulted in significantly higher F1 scores than unimodal conditions, except when the target stimulus was auditory in keyresTask (Figure 6B). With respect to the response type, keyresTask outperformed countTask under the uniA, uniV, and croV conditions, indicating different effects depending on the stimulus combination (Figure 6C). These results suggest that the highest classification performance was achieved using high-frequency auditory and low-frequency visual stimuli. Moreover, keyresTask enabled more accurate classification than countTask.

#### 3.3.2 Effects of polarity and number of templates

**Figure 7:**
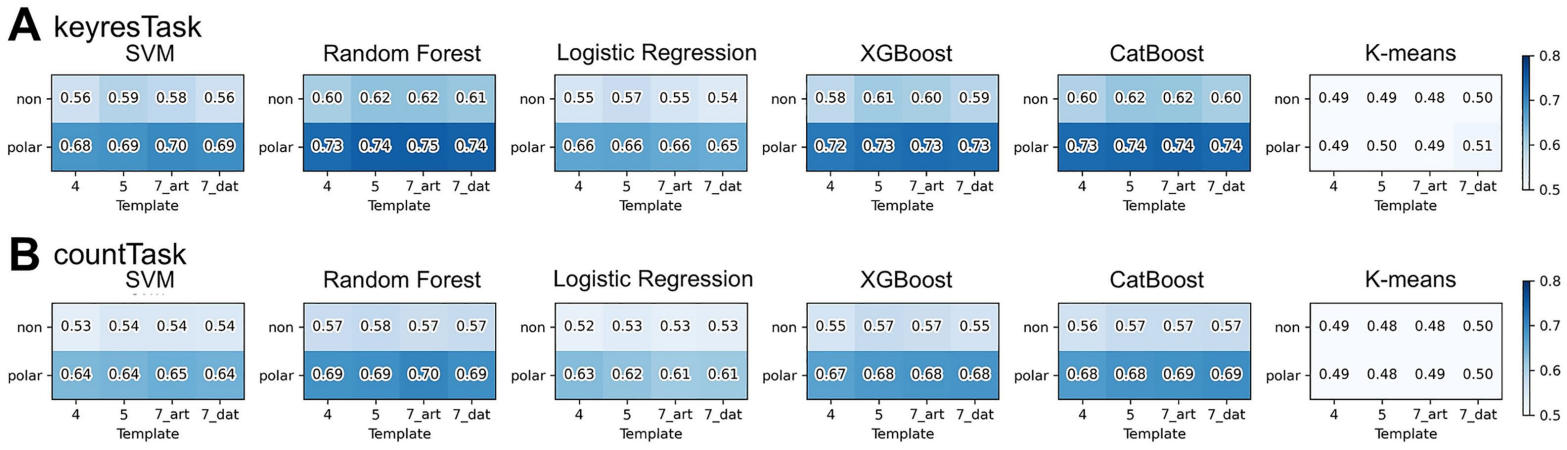
Effects of the number of templates and polarity consideration on classification performance. **(A)** Participant-averaged F1 scores for the keyresTask, and **(B)** Participant-averaged F1 scores for the countTask. Rows represent polarity conditions, and columns indicate the number of templates. This figure illustrates how polarity considered labeling (polar) generally improved classification performance compared to polarity-ignored labeling (non), whereas varying the number of templates had only a limited effect.

Figure 7 presents a matrix of participant-averaged F1 scores across conditions with and without polarity and across different numbers of templates. All five models, excluding K-means, yielded higher classification scores when polarity-considered templates were used.

**Figure 8:**
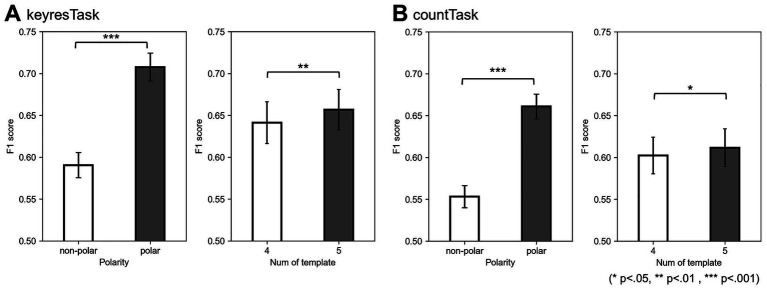
Effects of polarity and number of templates on F1 scores for the keyresTask **(A)** and countTask **(B)**. Error bars represent 95% confidence intervals across participants. In the keyresTask, polarity-considered labeling (polar) performed significantly better than polarity-ignored labeling (non-polar), and using five templates yielded significantly higher F1 scores than using four. In the countTask, only the main effect of polarity reached statistical significance.

An ANOVA was conducted using the average F1 scores from the five classification models (excluding K-means) to examine the effects of polarity and the number of template clusters, limited to cluster sizes of four and five (Figure 8). The interaction between cluster number and polarity was observed in keyresTask [*F*(1, 19) = 4.78, *p* < 0.05, $\eta_{p}^{2}\;$ = 0.20], but not in countTask [*F*(1, 19) = 2.74, *p* = 0.11, $\eta_{p}^{2}$ = 0.13]. Adding polarity significantly improved classification accuracy in both tasks [keyresTask: 19.8% improvement, *F*(1, 19) = 190.28, *p* < 0.001, $\eta_{p}^{2}$ = 0.91; countTask: 19.5% improvement, *F*(1, 19) = 290.26, *p* < 0.001, $\eta_{p}^{2}\;$ = 0.94]. Regarding the number of clusters, performance was significantly higher with five clusters compared to four [keyresTask: *F*(1, 19) = 11.09, *p* < 0.01, $\eta_{p}^{2}\;$ = 0.37; countTask: *F*(1, 19) = 4.59, *p* < 0.05, $\eta_{p}^{2}$ = 0.20].

### 3.4 Temporal differences in microstate label distributions

**Figure 9:**
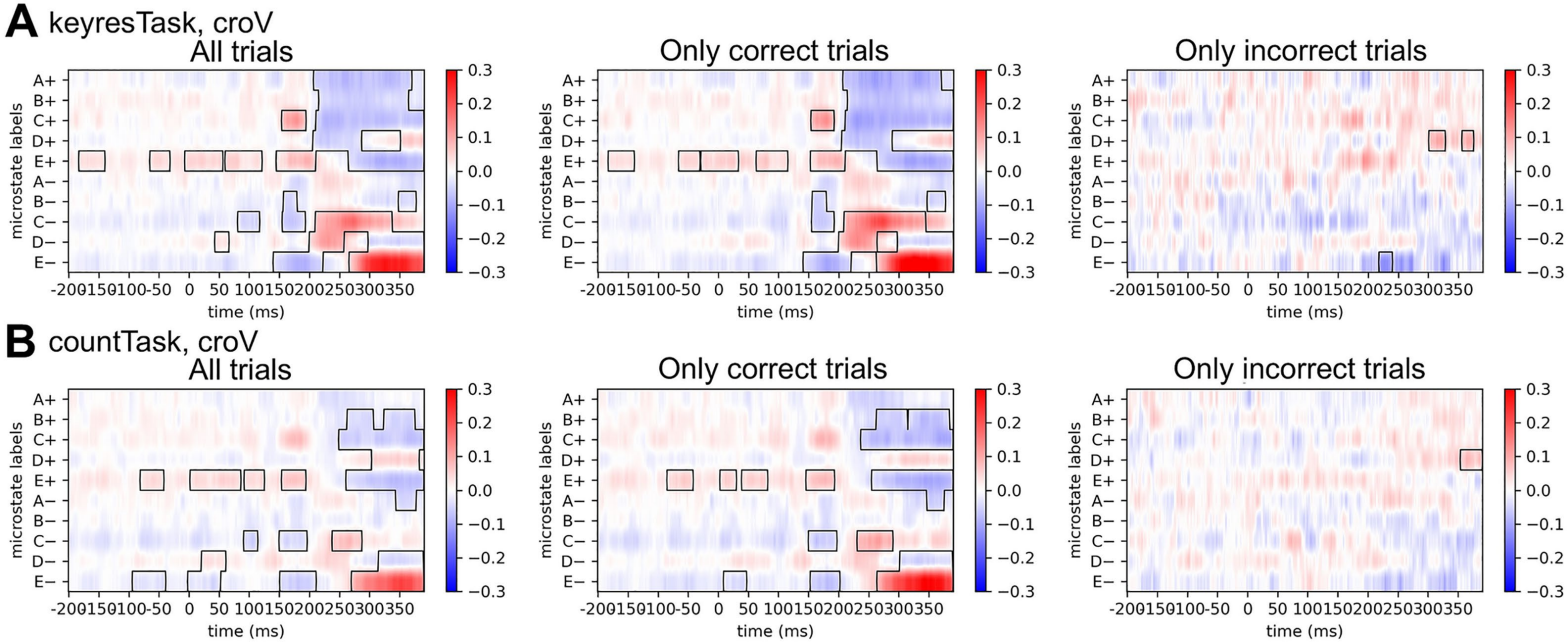
Differences in averaged microstate label distributions for the croV condition, computed as the mean distribution for target stimuli minus that for standard stimuli. **(A)** Results for the keyresTask. **(B)** Results for the countTask. The left panel represents results based on all trials, the middle panel includes only trials correctly classified by the random forest model, and the right panel includes only incorrectly classified trials. Regions with significant differences across participants are outlined with black contours. The color scale represents differences in microstate occurrence rates (target − standard): positive values (red) reflect greater occurrence in target stimuli, whereas negative values (blue) reflect greater occurrence in standard stimuli. In the left and middle panel cases, significant differences were observed at approximately 150 ms and 300 ms. By contrast, in the right panel, a significant difference in microstate D + emerged that was not present during the pre-stimulus period or at other time points.

To identify temporal regions where microstate label distributions differ between standard and target stimuli, time-resolved statistical comparisons based on the procedure described in Section 2.5 are shown in Figure 9. In the croV condition of the keyresTask, microstates A+, B+, and C+ showed significantly higher average label values for standard stimuli in the 200–350 ms range. Additionally, microstate D+ exhibited a significant increase in standard stimuli at approximately 250 ms, and E+ showed a similar pattern near 300 ms. By contrast, microstate E+ showed significantly higher values for target stimuli at approximately 200 ms, whereas C− (250–300 ms), D− (approximately 250 ms), and E− (300–350 ms) also favored the target condition. In countTask, no significant differences were observed for microstates A+, C−, or D−, in contrast to keyresTask. Only microstate B+ showed a significant difference near 350 ms, with higher values for standard stimuli.

When restricting the analysis to trials correctly classified by the Random Forest model, the keyresTask revealed significantly higher values for target stimuli in microstate E− from 0 to 100 ms and for standard stimuli in microstate C− from 50 to 100 ms. In countTask, a significant increase in microstate D+ (approximately 350 ms) was observed for target stimuli when using all trials; however, this difference disappeared when limiting the analysis to correctly classified trials.

### 3.5 Reconstructed topographies by microstate weights

**Figure 10:**
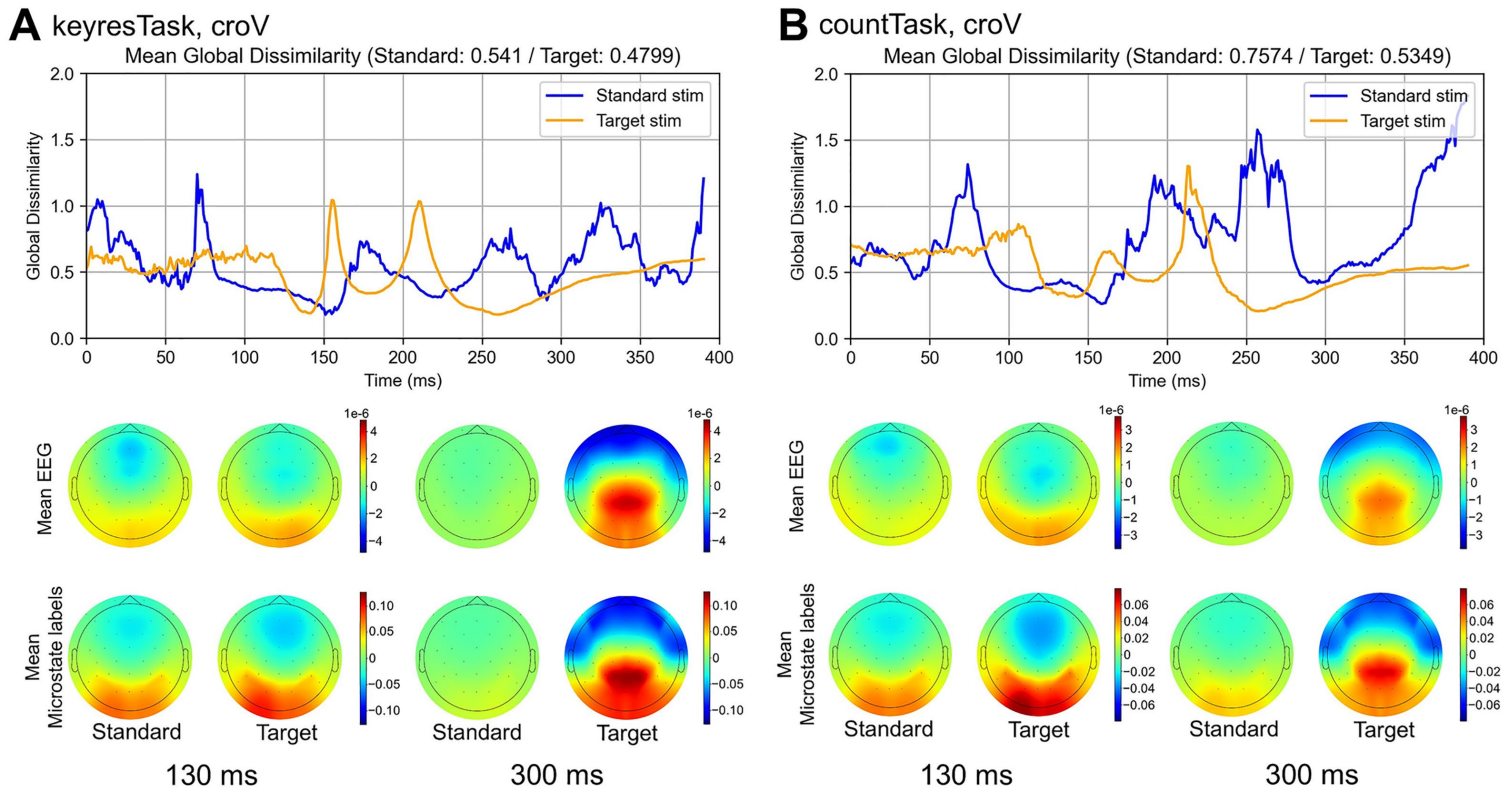
Top: Global dissimilarity (DISS) between the grand-averaged EEG and the reconstructed EEG obtained by summing the products of the mean microstate labels and their corresponding templates in the croV condition, shown for both the keyresTask **(A)** and the countTask **(B)**. DISS is a scale-independent measure of topographic dissimilarity based on normalized voltage distributions across electrodes, ranging from 0 (identical maps) to 2 (maps with inverted polarity), thus providing an estimate of information loss with respect to topographic similarity. In both tasks, DISS values for the target stimuli were closer to 0 than those for the standard stimuli, indicating a more faithful reconstruction of the target-related EEG topographies. Bottom: Topographic maps at 130 ms and 300 ms, illustrating the original and reconstructed EEG for comparison.

Topographic maps were reconstructed at each time point using a weighted sum of the microstate templates, as described in Section 2.6, and the results are shown in Figure 10. In the croV condition of the keyresTask, the average DISS was 0.712 for standard stimuli and 0.569 for target stimuli. In the countTask, the average DISS was 0.893 for standard stimuli and 0.605 for target stimuli. Across the entire 0–400 ms window, the DISS values for target stimuli remained consistently lower and less variable than those for standard stimuli. In both tasks, the standard stimuli yielded higher mean DISS values, with the difference being more pronounced in the countTask. The topographic maps at 130 and 300 ms (Figure 10, bottom) illustrate the original and reconstructed EEG for comparison.

## 4 Discussion

This study had three main objectives: (I) evaluate the classification performance of P300 responses using microstate labeling, which differs from conventional methods; (II) explore the potential improvement in performance using polarity-considered microstate labeling; and (III) examine the relationship between classification performance and different experimental conditions in an oddball paradigm using microstate labeling. The discussion is structured based on these objectives.

### 4.1 P300 classification performance using a non-conventional labeling method

Unlike conventional methods, such as SWLDA, which have demonstrated over 90% classification accuracy (Krusienski et al., 2008), the present study employed one-dimensional microstate label sequences for classification. Among the five models, SVM, Random Forest, Logistic Regression, XGBoost, and CatBoost achieved F1 scores above the chance level. Logistic Regression can be regarded as linear classifier, whereas SVM can operate as either a linear or a nonlinear classifier depending on the kernel. In this study, kernel options for SVM were restricted to linear, rbf, and sigmoid; when the linear kernel was selected, SVM was regarded as a linear classifier. Tree-based models, such as Random Forest, XGBoost, and CatBoost, which are nonlinear classifiers, showed consistently high and stable AUC values, suggesting that these models are best suited for classification based on EEG microstate labeling. The superior performance of nonlinear models compared with linear models suggests that the distribution of microstate labels may exhibit nonlinear characteristics. By contrast, the unsupervised clustering method K-means failed to exceed chance level performance. This outcome can be attributed to two factors. First, as an unsupervised approach, K-means does not exploit label information and relies solely on Euclidean distance for cluster formation, making it inherently disadvantaged in supervised classification tasks. Second, the one-dimensional integer-valued microstate sequences starting from 0 did not exhibit clear cluster structures aligned with the given labels, limiting the applicability of clustering-based methods. These results emphasize that the distribution of microstate labels cannot be adequately captured by simple clustering assumptions. Rather, it reflects complex nonlinear dynamics, which is consistent with the superior performance of nonlinear supervised models observed here.

Figure 2 shows that labels C and E frequently appeared after stimulus onset across all tasks, but no single label dominated, indicating variability across conditions. Tarailis et al. (2024) associated microstate C with internal processes, such as self-reflection and autobiographical memory, whereas microstate E is linked to emotional processing and interoceptive attention. The co-occurrence of both labels suggests that the oddball task evokes parallel internal cognitive and bodily responses, resulting in dynamic transitions in brain states and a nonlinear structure in label sequences. Even in standard stimulus trials, label sequences occasionally resemble those of target stimuli, further supporting the nonlinearity of label distribution. Although the classification accuracy of this method reached approximately 80% at best under the croV condition, which was lower than that reported for conventional methods, the use of label sequences enabled greater interpretability of the spatial dynamics in EEG, as discussed in Section 3.1.

### 4.2 Potential performance improvement through polarity-considered microstate labeling

**Figure 11:**
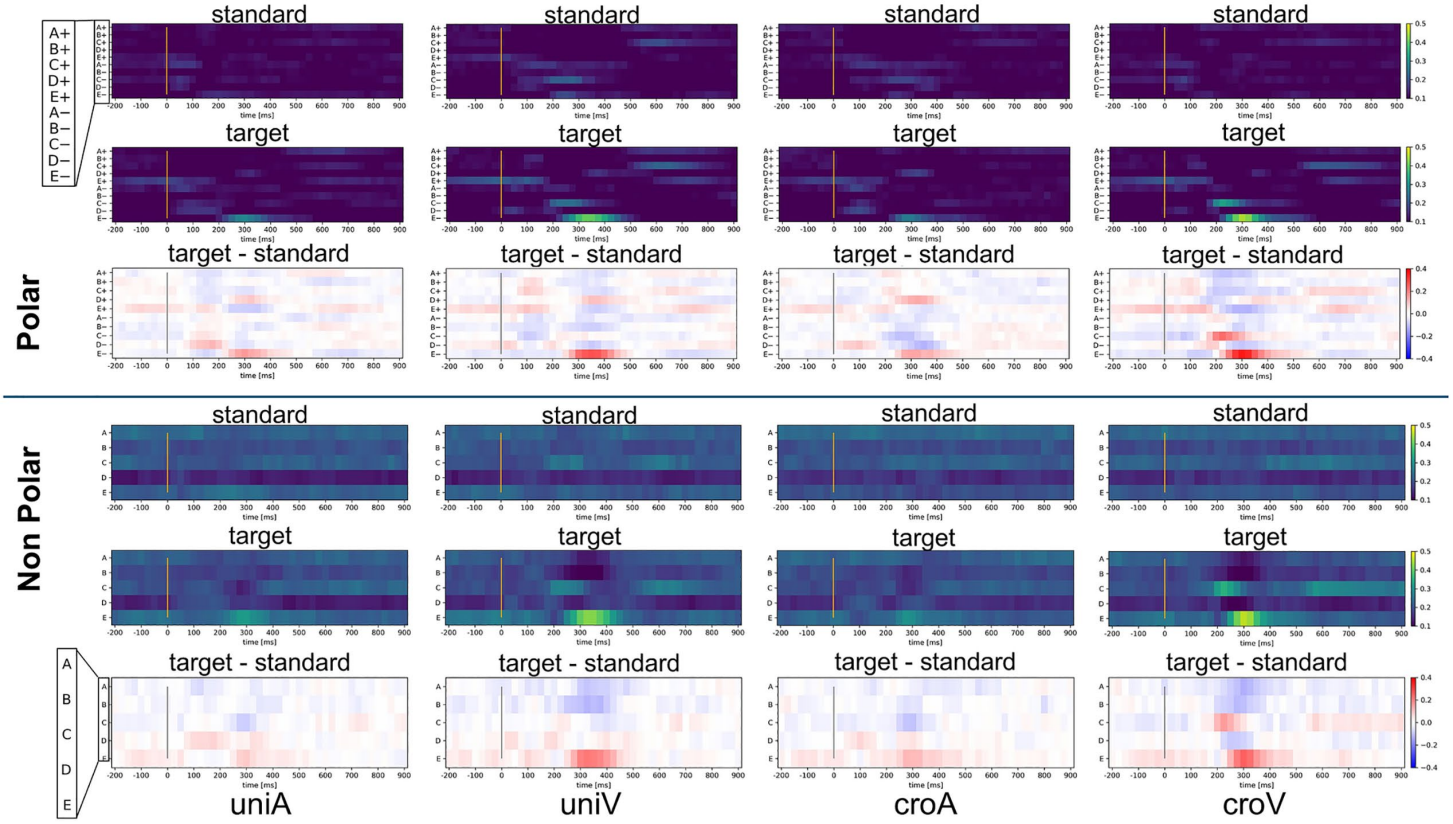
Polarity-related differences in EEG microstate occurrence rate by keyresTask. In the “standard” and “target” panels, brighter colors indicate higher EEG microstate occurrence rates. In the “target-standard” panels, red denotes higher occurrence rates for the target condition, blue denotes higher occurrence rates for the standard condition, and white indicates no difference between the two. This figure illustrates how preserving polarity provides a finer granularity of label space, revealing distinct temporal patterns that correspond to N200- and P300-related microstates. Each column panel represents a different stimulus condition.

This study adopted a labeling method that preserved the polarity of EEG microstates. Incorporating polarity into the templates resulted in statistically significant improvements in the classification accuracy compared with the polarity-ignored condition. To explore how the inclusion of polarity affects label information, we evaluated the time course of the occurrence rates averaged across participants for keyresTask (Figure 11) following the same approach as in Section 2.3. The polarity-considered microstate labeling produced a label space with approximately twice the granularity compared to polarity-ignored labeling, effectively distributing the information across a broader scale and potentially enhancing the classification performance. Additionally, the underlying neural dynamics can be interpreted as follows: following the onset of a target stimulus, positive frontal topographies (C+ and E+) emerged within 0–200 ms, followed by D− at 200–300 ms and E− at 300–400 ms. Among these, D− in the N200 time window and E− in the P300 time window exhibit spatial topographies similar to those of the N200 and P300 components reported by Mahini et al. (2024). Mahini et al. (2024) stated that “Cluster 2, which represents the P300 component, was most frequently observed between 280 and 540 ms,” indicating the time window with the highest spatial correlation for the P300 cluster derived via clustering. Although the results were averaged across participants and covered a slightly narrower time window, they exhibited comparable temporal patterns. The dynamic changes observed included initial attentional switching and early sensory response within 0–200 ms, internal re-evaluation (approximately 200–300 ms), and evaluative or decision-related processes between 300 and 400 ms, which supports the interpretation that P300 is closely linked to the E− label. However, when polarity is ignored, this nuanced transition is lost, and these distinct states collapse into a single E label, obscuring their interpretability. This likely contributed to the observed drop in classification performance under polarity-ignored conditions. Mahini et al. (2024) also observed that polarity-inverted topographies enable the formation of stable clusters for N200 and P300, supporting the notion that polarity enhances classification precision. Similarly, Kashihara et al. (2025a) pointed out that including polarity is essential for accurately capturing the continuous topological structure of the EEG state space and for maintaining the inherently smooth and directional trajectories of microstate transitions. Our findings align with this perspective, suggesting that polarity information is crucial for approximating nonlinear, continuous brain activity dynamics in a discrete label space.

### 4.3 Correspondence between classification performance and oddball task conditions via microstate labeling

The effects of three experimental factors—(i) the modality of infrequent stimuli (auditory or visual), (ii) combinations of frequent and infrequent stimuli (unimodal or cross-modal), and (iii) the type of response to infrequent stimuli (key-response task or count task)—were evaluated and are presented in Section 3.3.1.

First, regarding factor (i), stimulus modality—visual stimuli showed significantly higher classification accuracy than auditory stimuli (except in the cross-modal condition of the countTask). This suggests that visual stimuli are more effectively classified using the proposed method. These results are consistent with those of Katayama and Polich (1999) and Polich and Heine (1996). Therefore, this result may reflect occipital neural activity induced by visual input, which strongly contributes to spatial distribution patterns and forms brain states that are easier for the classifier to distinguish.

Second, for factor (ii), the combination of stimulus modalities—the cross-modal condition involving target visual stimuli in the keyresTask—yielded significantly higher accuracy than the unimodal condition, whereas no significant differences were observed in other tasks. This indicates that the combination of modalities is effective only when the task involves rapid motor responses, such as in keyresTask. In this study, several findings aligned with the traditional P300 literature. The improved classification accuracy observed in some cross-modal conditions compared with unimodal conditions may be explained by Calvert et al. (2000), who reported that multimodal integration imposes an additional neural processing load. The keyresTask, which requires quick responses, may have been more influenced by this transient high cognitive load than the countTask, in which no reaction-time constraint was imposed.

Finally, for factor (iii), response type to target stimuli, a significantly higher classification accuracy was observed in the keyresTask than in the countTask in the uniA (auditory only) and croV (standard auditory, target visual) conditions. This indicates that task differences emerged only when the standard stimulus was auditory. These findings are consistent with those reported by Brázdil et al. (2003) and Kotchoubey (2014). One likely explanation for this task-related difference is that keyresTask requires participants to respond quickly, whereas countTask does not, resulting in varied response times across trials. Additionally, the task differences appearing only when the standard stimulus was auditory may be due to the fact that auditory stimuli tend to elicit faster responses than visual stimuli. In a study by Shelton and Kumar (2010), the mean reaction time for auditory stimuli was reported to be approximately 284 ms, which is significantly shorter than the 331 ms observed for visual stimuli. This finding supports the interpretation that keyresTask, which involves immediate responses, yields significantly higher classification accuracy under auditory-dominant conditions.

### 4.4 Temporal specificity of microstate labeling

The temporal differences in microstate label distributions between standard and target stimuli corresponded closely with well-known ERP components observed in the oddball task, particularly N200 and P300. At approximately 250 ms, microstates C− and D− were more prominently observed in response to target stimuli, likely reflecting enhancement of the front-central N200 component. According to Tomé et al. (2015), the N200b subcomponent is associated with “controlled detection of stimulus changes” and “phonological categorization” and is commonly described as reflecting the classification or categorization of deviant stimuli. The present results align with these prior findings, as the N200-related microstates were more strongly expressed for the target stimuli. Following this, approximately 300 ms post-stimulus, microstate E− was dominant for the target stimuli, whereas A− and C− appeared more frequently for standard stimuli. The P300 component is linked to context updating and attentional switching in response to deviant stimuli that differ from the standard (Donchin, 1981; Polich, 2007). Therefore, the predominance of microstate E − between 300—350 ms in the target condition can be interpreted as a neural correlation of attentional reallocation. Conversely, the standard condition elicited less P300-related activity and showed prolonged occupancy of earlier microstates, such as A+, B+, and C+. This suggests that, in the absence of target detection, brain activity may remain in earlier sensory processing states. Thus, the observed microstate transitions clearly reflect neural progression from N200 to P300 processing when a target stimulus appears.

With respect to response type, a greater number of microstate differences were observed in the keyresTask than in the countTask. Salisbury et al. (2001) reported that button-press responses reduce P300 amplitude and alter its scalp topography compared with silent-count conditions, likely owing to the contribution of motor-related activity. By contrast, in the present study, the keyresTask showed a larger amplitude around 300 ms than the countTask, but the distribution was more frontally oriented. These findings suggest that response format does not necessarily produce consistent effects on P300 amplitude; however, it may influence scalp topography in a manner broadly consistent with the involvement of motor-related activity.

Notably, when analyzing only those trials that were correctly classified by the Random Forest model, some differential microstate activity emerged at earlier latencies (0–100 ms). In the key-press task, target stimuli showed higher occupancy of microstate E–, even within the first 100 ms of stimulus onset (with standard stimuli conversely higher in microstate C– at 50–100 ms). According to Tomé et al. (2015), target stimuli elicit larger N100 components than standard stimuli do, reflecting the early sensory acquisition of the stimulus. Therefore, it is plausible that the correctly classified trials were those in which such early sensory differentiation, similar to the N100 component, was more pronounced. On the other hand, differences observed in the incorrectly classified trials tended to be larger than those in the correctly classified ones. One possible explanation is that misclassified responses may reflect atypical or inconsistent microstate dynamics, which exaggerate the contrast between standard and target stimuli. In addition, the imbalance in the number of correct and incorrect trials may have influenced the statistical comparisons, making the differences appear larger in the incorrect condition.

### 4.5 Interpretation of composite topographies

In Section 3.5, the EEG topography was reconstructed using the weighted sum of the microstate templates, and the DISS between the reconstructed and average EEG topographies was calculated. The results demonstrated that the DISS values varied depending on both stimulus and response types. According to Koenig et al. (2002), four to seven microstate maps can explain over 70% of the total EEG variance. By contrast, in this study, the average DISS values were approximately 0.8 for standard stimuli and approximately 0.6 for target stimuli. Based on the definition of DISS, where 0 represents complete similarity and 2 represents complete dissimilarity, these results indicate that the target stimulus condition exhibited a relatively higher degree of similarity than the standard stimulus condition, suggesting that the reconstructed topographies more closely aligned with the original EEG topographies in the target stimulus condition. Furthermore, time points around 130 ms and 300 ms correspond to periods during which N200 and P300 related components are typically observed. At these time points, both standard and target stimuli exhibited similarly low DISS values, representing time points at which the reconstructed and original EEG topographies showed a high degree of spatial correspondence (Figure 10). This finding suggests that microstate-based topographic reconstruction captures spatial structures that are not limited to specific ERP components but are stable across different temporal stages of information processing. With respect to response type, lower DISS values were observed in the keyresTask than in the countTask for both standard and target stimuli. In particular, the standard stimuli condition in the countTask showed relatively high DISS values and greater variability in the reconstructed topographies. This result suggests that conditions that lack overt behavioral responses may yield less stable spatial structure of stimulus-induced neural responses.

**Figure 12:**
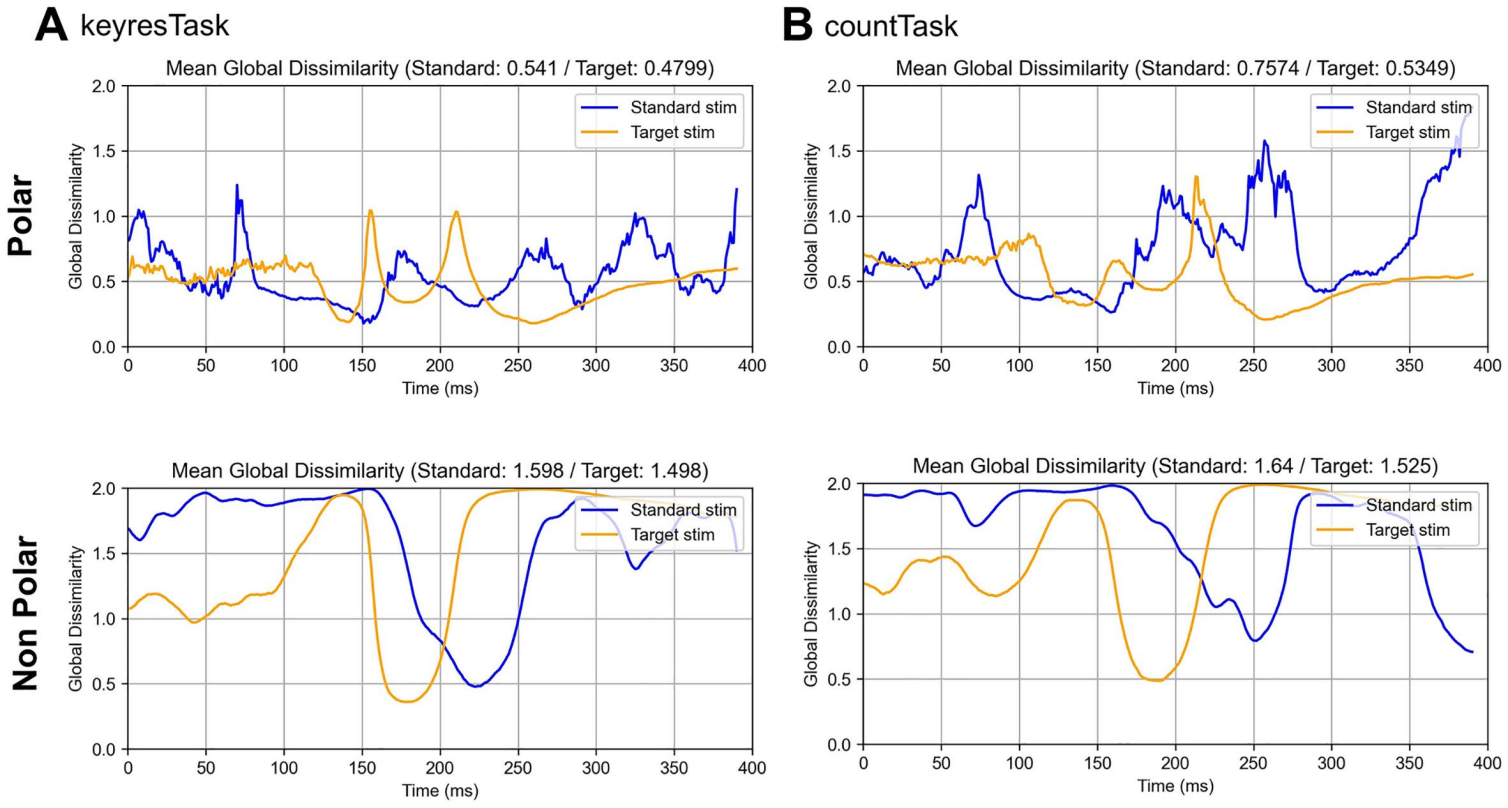
Temporal changes in global dissimilarity (DISS) between grand-averaged EEG topographies and reconstructed EEG topographies obtained using microstate labeling, with and without polarity consideration. Top: Polarity-considered condition for the keyresTask **(A)** and countTask **(B)**. Bottom: Polarity-ignored condition. With polarity considered, both target and standard stimuli yielded DISS values closer to 0, indicating that the reconstructed topographies more closely resembled the original EEG maps. By contrast, in the polarity-ignored condition, DISS values approached 0 only approximately 150–200 ms for the target condition and 180–250 ms for the standard condition. Outside these windows, DISS values generally exceeded 1, reflecting polarity-inverted topographies and corresponding information loss.

Figure 12 illustrates the temporal progression of DISS for reconstructed EEG topographies obtained using either the original microstate templates alone or polarity-considered microstate templates that additionally incorporated polarity-inverted versions of the original templates. When polarity was not distinguished, DISS values were consistently higher across all conditions, indicating reduced spatial correspondence between the reconstructed and original EEG topographies. When only the original microstate templates were used, topographies with opposite polarities were treated as the same state, leading to increased mixing of inverse-polarity patterns and consequently higher DISS values. By contrast, when polarity-considered templates were used, topographies with opposite polarities were represented as distinct states, thereby reducing confusion during reconstruction and preserving spatial structures more closely aligned with the original EEG signals. These results indicate that considering polarity in microstate analysis preserves the sign of the electrical potential as well as a representational space that distinguishes distinct brain states. Moreover, this pattern is consistent with the classification results shown in Figure 7. Polarity-considered microstate labeling appears to construct a more separable EEG topographic state space, thereby providing feature representations that are more discriminative for classification. In particular, topographies associated with N200- and P300-related components are characterized by polarity-dependent spatial distributions, suggesting that distinguishing polarity contributes to the preservation of physiologically meaningful EEG structures.

### 4.6 Real-time responsiveness

In this study, standard and target stimuli were classified offline using one-dimensional information derived from microstate labeling, whereby each EEG segment was assigned the template label showing the maximum spatial correlation between the precomputed microstate templates and task-related EEG data. To evaluate the latency for real-time implementation, we examined the processing speed of the proposed pipeline. A classification model was trained in advance using supervised classifiers, including SVM, Random Forest, Logistic Regression, XGBoost, and CatBoost, whereas the unsupervised K-means approach was excluded from real-time evaluation. For raw EEG data, preprocessing steps included resampling to 1,000 Hz, bandpass filtering between 1 and 45 Hz, and applying a common average reference. Epochs were extracted from −0.2 s to 0.4 s relative to stimulus onset, followed by baseline correction using the pre-stimulus interval (−0.2 to 0 s). After microstate labeling, the extracted features were input into the trained model to generate predictions. All computations were performed on a Windows environment [OS: Windows 11; CPU: Intel(R) Core(TM) i7-11800H; RAM: 16 GB] using Python.

Processing throughput (bps) was quantified for two stages: (1) from raw data loading to prediction, and (2) from microstate labeling to prediction. Because offline datasets were used, the data-loading stage required additional processing time. The results are presented in Supplementary Table 2. However, the throughput from microstate labeling to prediction exceeded 1.5 Mbps across all models, corresponding to a latency of approximately 0.25 s. Although model training requires substantial computation time in advance, predictions can subsequently be obtained within approximately 0.7 s of stimulus presentation, suggesting the feasibility of real-time implementation.

In addition, some previous P300-related studies preserve low-frequency components beginning at 0.1 Hz and applied baseline correction. Therefore, we evaluated alternative preprocessing pipelines using resampling to 1,000 Hz, a 0.1–45 Hz bandpass filter, and common average reference, with and without baseline correction, followed by comparison of their classification results with those obtained in the present study. Although applying baseline correction under the 0.1–45 Hz filtering condition modestly improved classification performance, the overall improvement remained inferior to that achieved with the 1–45 Hz filtering strategy. These findings indicate that suppressing of slow drifts and artifacts plays a more critical role than baseline correction alone in enhancing classification performance based on microstate labeling. Specifically, the subject-averaged F1 score using Random Forest (keyresTask, uniV) was 0.700 for 0.1–45 Hz without baseline correction, 0.768 for 0.1–45 Hz with baseline correction, and 0.841 for 1–45 Hz without baseline correction.

### 4.7 Limitations and future directions

Despite these findings, this study had several limitations. First, because the microstate templates used in this study were derived from the LEMON dataset (Babayan et al., 2019), individual variability in brain state dynamics may have been overlooked. Utilizing subject-specific templates could further enhance classification accuracy. To address this limitation, task-specific microstate templates were additionally constructed following a procedure analogous to that applied to the LEMON-based templates. For each experiment and task, artifact-corrected continuous EEG data were used. From each participant, 2,500 GFP peak maps were randomly selected, with a minimum peak-to-peak interval of 10 ms. As each task (keyresTask and countTask) included 20 participants, this resulted in 50,000 maps per task for clustering. These maps were clustered using a modified K-means algorithm to derive five representative topographies. To incorporate polarity, each map was multiplied by −1 and included as an additional template, resulting in a total of 10 task-based templates per condition. Correlation analysis between the task-based templates and LEMON-based templates revealed that several templates closely resembled canonical classes (e.g., A, B, and C). The results are presented in Supplementary Figure 2. By contrast, templates exhibiting pronounced hemispheric asymmetry or substantial differences between parietal and other regions appeared to reflect task-specific patterns. However, because the derived templates were not entirely consistent across tasks, the use of LEMON-based templates may serve as a form of normalization by providing a stable reference framework across experimental conditions.

Second, because this study focused on offline analysis using multiple classification models, the performance of the proposed method in real-time BCI systems remains unverified. Future studies should explore the practical implementation of the proposed method for real-time BCI systems, such as the P300 speller proposed by Farwell and Donchin (1988). Moreover, direct comparison of the proposed approach with state-of-the-art waveform-based P300 methods (e.g., Riemannian geometry or xDAWN + SWLDA) was not performed. Recent microstate-based advances such as continuous decoding or deep learning integration, were also not addressed. These remain important directions for future work.

Third, the selection of temporal window parameters may also limit the generalizability of the present findings. Specifically, we evaluated combinations of time-window lengths (10, 50, and 100 ms) and step rates (10, 25, 50, 75, and 90%). The results are presented in Supplementary Figure 3. In Section 2.4.1, a 100 ms window with a 25% step rate was provisionally adopted. However, for subsequent classification analyses, we selected the combination that yielded the highest performance (10 ms window with a 10% step rate). Optimal parameter settings may vary depending on the EEG recording system, sampling rate, or task characteristics. Therefore, further validation under different experimental conditions remains necessary to determine the robustness of these parameter choices.

In conclusion, this study demonstrated that polarity-considered EEG microstates significantly improved the classification accuracy of infrequent stimuli in an oddball paradigm. Across multiple models, excluding K-means, incorporating polarity into the labeling process consistently yielded higher performance than polarity-ignored labeling, whereas varying the number of templates had only a limited effect. Furthermore, the cross-modal condition with infrequent visual stimuli in the key-response task achieved the highest accuracy, indicating that both stimulus modality and response type play important roles in microstate-based classification. These findings indicate that integrating polarity-considered microstate labeling with optimized experimental conditions enhances classification performance and underscores its potential for BCI applications, including P300 spellers.

## Data Availability

The original contributions presented in the study are included in the article/Supplementary material, further inquiries can be directed to the corresponding author.
