# Supplementary material for: Polarity-considered EEG microstates improve classification accuracy of oddball stimulus
Source: Front Hum Neurosci. 2026 Mar 18;20:1712380. doi: 10.3389/fnhum.2026.1712380 (PMC13038975; doi:10.3389/fnhum.2026.1712380)
Supplement: Supplementary file 1 [file Data_Sheet_1.docx]

Supplementary Material

# Supplementary Figures and Tables

**
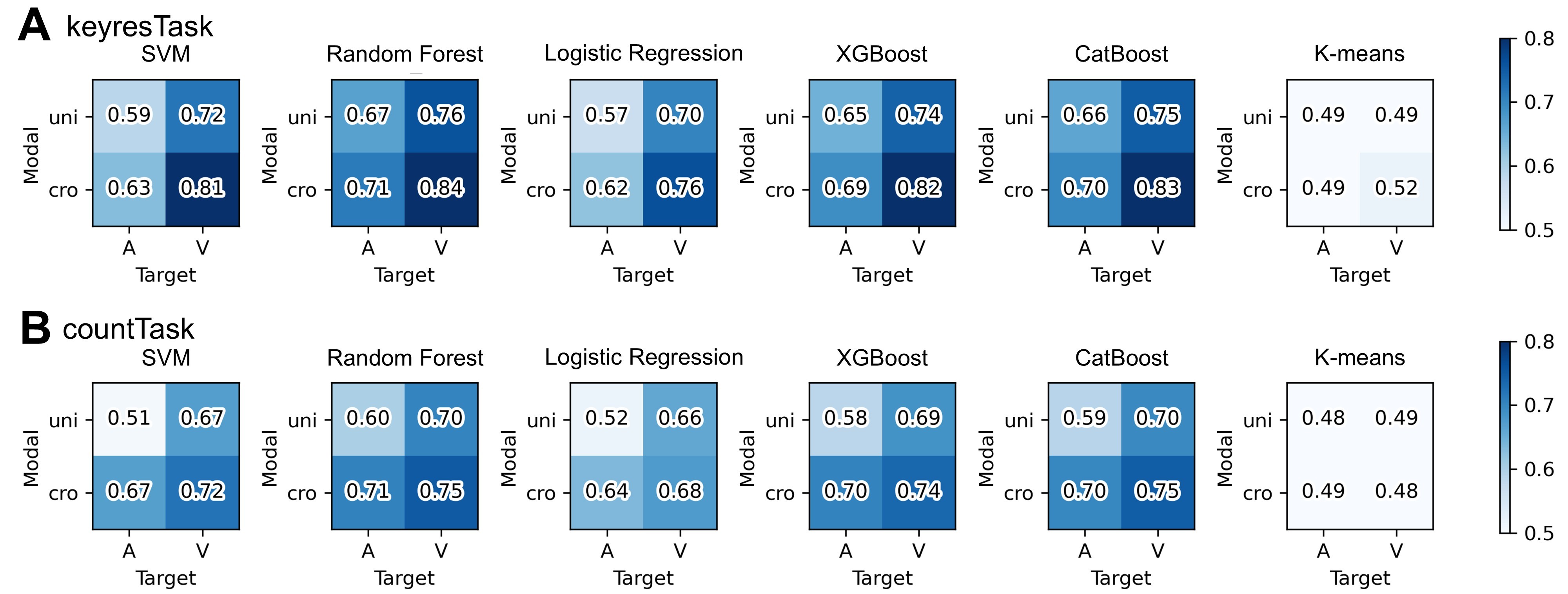
**

**Supplementary Figure 1.** Target modal matrix by keyresTask (A) and countTask (B). The color scale represents participant-averaged F1 score across stimulus conditions. This figure visually demonstrates that the participant-averaged F1 score was lowest for uniA (lighter colors) and highest for croV (darker blue). Since the chance level is 0.5, it can also be seen that K-means remained approximately at this level.


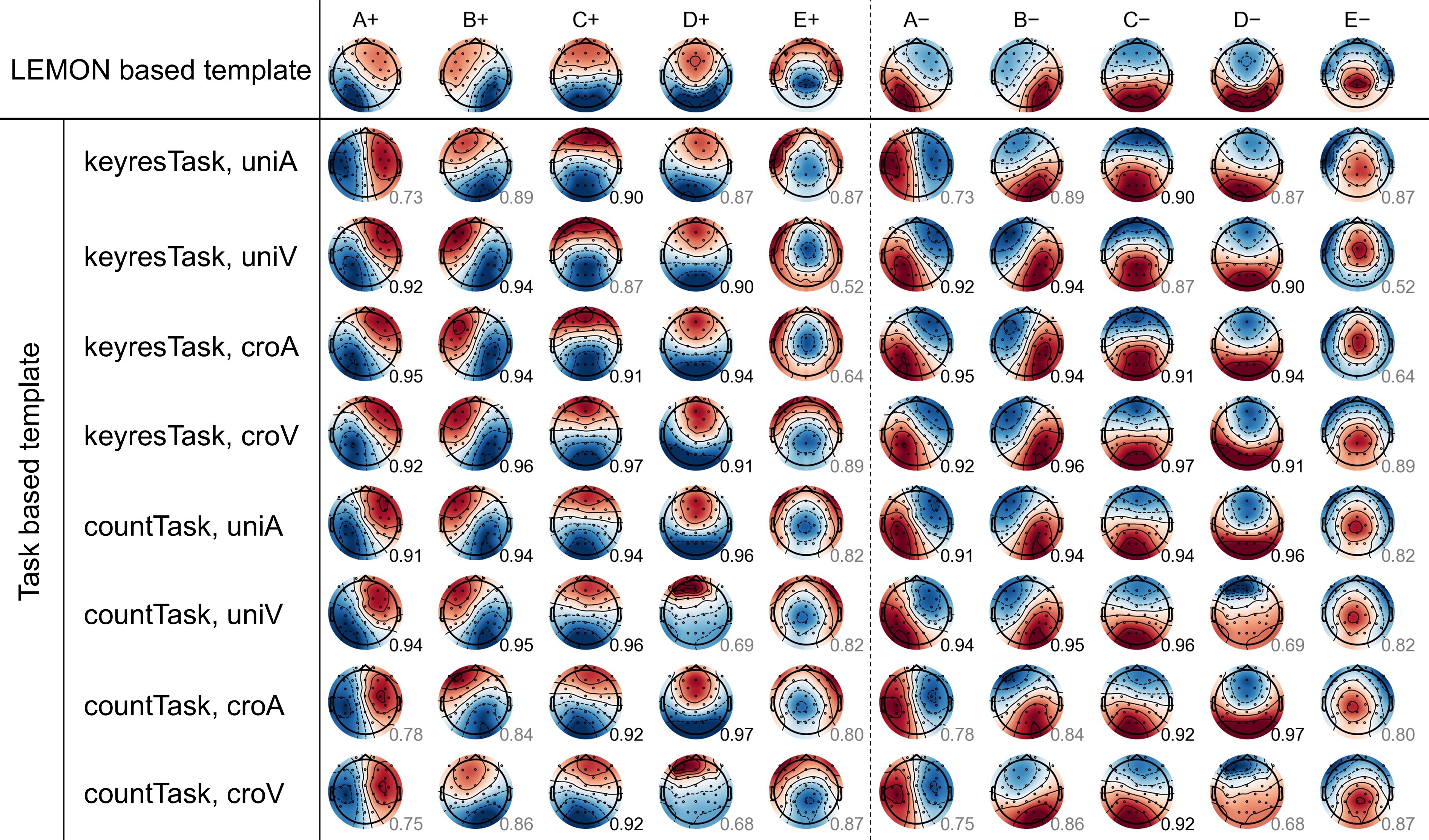


**Supplementary Figure 2.** Comparison of LEMON-based and task-specific microstate templates. The top row shows canonical templates from the LEMON dataset (A+, B+, …, E−). Task-specific templates were generated separately for each condition (keyresTask: uniA, uniV, croA, croV; countTask: uniA, uniV, croA, croV) using modified K-means clustering of GFP peak maps with polarity inversion. Several templates resembled canonical classes, while others reflected task-specific topographies.


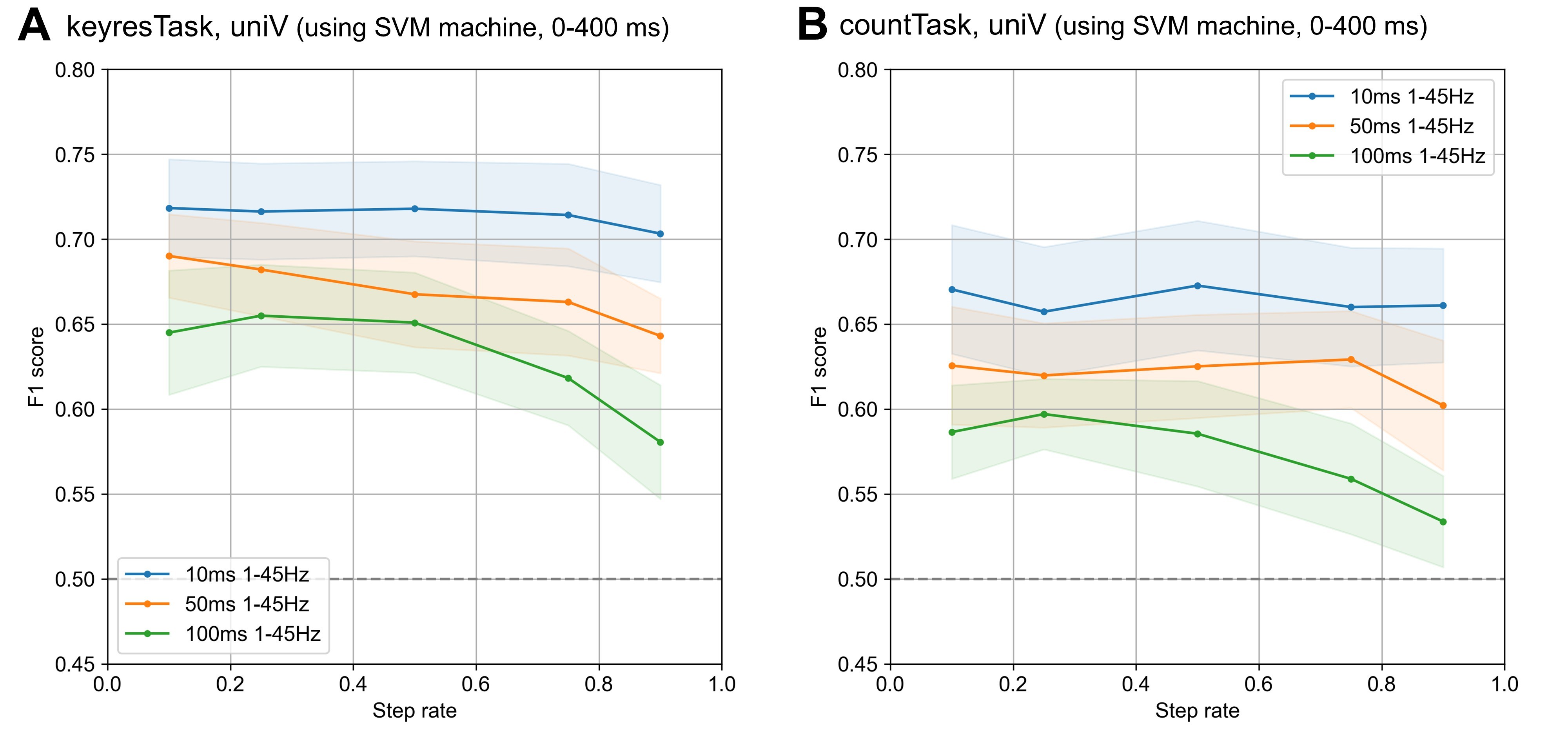


**Supplementary Figure 3.** Classification performance across different temporal window lengths and step rates. Classification performance (mean F1 score across participants) is shown for combinations of time-window lengths (10, 50, and 100 ms) and step rates (10%, 25%, 50%, 75%, and 90%). The left panel presents results for the uniV condition in the keyresTask, and the right panel presents results for the uniV condition in the countTask. Shaded regions indicate 95% confidence intervals across participants. Window lengths are color-coded as follows: 10 ms (blue), 50 ms (orange), and 100 ms (green). A 100 ms window with a 25% step rate was provisionally adopted in Section 2.4.1, whereas the combination yielding the highest classification performance (10 ms window with a 10% step rate) was selected for subsequent analyses.

**Supplementary Table 1.** Names of Python packages and hyperparameters used for classification models. From left to right, the columns indicate the name of classification model, the package name, and the hyperparameters tuned via grid search.

| Model | Package | Hyperparameters |
| --- | --- | --- |
| SVM | Scikit-learn | C: 0.01, 0.1, 1, 10, 100; Kernel type: linear, rbf, sigmoid; Gamma option: scale, auto, 1 |
| Random Forest | Scikit-learn | Number of estimators: 50, 100, 200; Maximum depth: None, 10, 20; Minimum samples split: 2, 5, 10 |
| Logistic Regression | Scikit-learn | Penalty: l1, l2; C: 0.001, 0.01, 0.1, 1, 10; Solver: liblinear, saga; Maximum iterations: 100, 500, 1000 |
| XGBoost | xgboost | Learning rate: 0.01, 0.1, 0.2; Maximum depth: 3, 5, 7; Number of estimators: 50, 100, 200; Subsample: 0.8, 1.0; Colsample by tree: 0.8, 1.0; Gamma: 0, 0.1, 0.2 |
| CatBoost | catboost | Iterations: 100, 200; Learning rate: 0.01, 0.1; Depth: 3, 5, 7; L2 leaf regularization: 1, 3, 5 |
| K-means | Scikit-learn | Number of clusters: 2; Initialization method: k-means++, random; Number of initializations: 10, 20, auto; Maximum iterations: 300, 500; Tolerance: 1e-4, 1e-5 |

**Supplementary Table 2.** Processing speed (bps) from offline data acquisition to prediction by machine learning. After data acquisition, the following preprocessing steps were applied: resampling to 1000 Hz, band-pass filtering (1–45 Hz), common average referencing, epoching from −0.2 s to 0.4 s relative to stimulus onset, and baseline correction using the mean value from −0.2 s to 0 s. Subsequently, microstate labeling was performed, and the resulting one-dimensional data were used for prediction with pre-trained machine learning models. The “Total” row indicates the processing speed (bps) from data acquisition to prediction, whereas the “Labeling–predict” row indicates the processing speed (bps) from microstate labeling to prediction. The machine learning models used were SVM, Random Forest, Logistic Regression, XGBoost, and CatBoost. Processing speed was measured excluding the training phase. All computations were performed on a Windows environment (OS: Windows 11; CPU: Intel(R) Core(TM) i7-11800H; RAM: 16 GB) using Python.

| (bps) | SVM | Random Forest | Logistic Regression | XGBoost | CatBoost |
| --- | --- | --- | --- | --- | --- |
| Total | 158$\times{10}^{3}$ | 159$\times{10}^{3}$ | 157$\times{10}^{3}$ | 157$\times{10}^{3}$ | 158$\times{10}^{3}$ |
| Labeling - predict | 1.57$\times{10}^{6}$ | 1.62$\times{10}^{6}$ | 1.66$\times{10}^{6}$ | 1.64$\times{10}^{6}$ | 1.62$\times{10}^{6}$ |
